# Supplementary material for: Soil Fungal Community Composition Correlates with Site-Specific Abiotic Factors, Tree Community Structure, and Forest Age in Regenerating Tropical Rainforests
Source: Biology (Basel). 2021 Oct 31;10(11):1120. doi: 10.3390/biology10111120 (PMC8614695; doi:10.3390/biology10111120)
Supplement: Supplementary file 1 [file biology-10-01120-s001.zip › biology-1425226-supplementary.pdf]

Richness and Composition of Soil Fungal Communities Correlate with Site-Specific Abiotic Factors, Tree Community Composition, and Forest Age in Regenerating Tropical Rainforests

Irene Adamo [1,2,3], Edgar Ortiz-Malavasi [4], Robin Chazdon [5], Priscila Chaverri [6,7], Hans ter Steege [1], József Geml [1,2,8]*

Supporting Information

Figure S1. (a) Mean Diurnal Range, Mean Annual Precipitation and Precipitation Seasonality in the two different regions. (b) Organic matter, estimated nitrogen release, pH and soil chemical elements for primary and secondary forest (SF) in the two regions, Piro and Mogos. The letters denote the significance difference (Tukey HSD test).

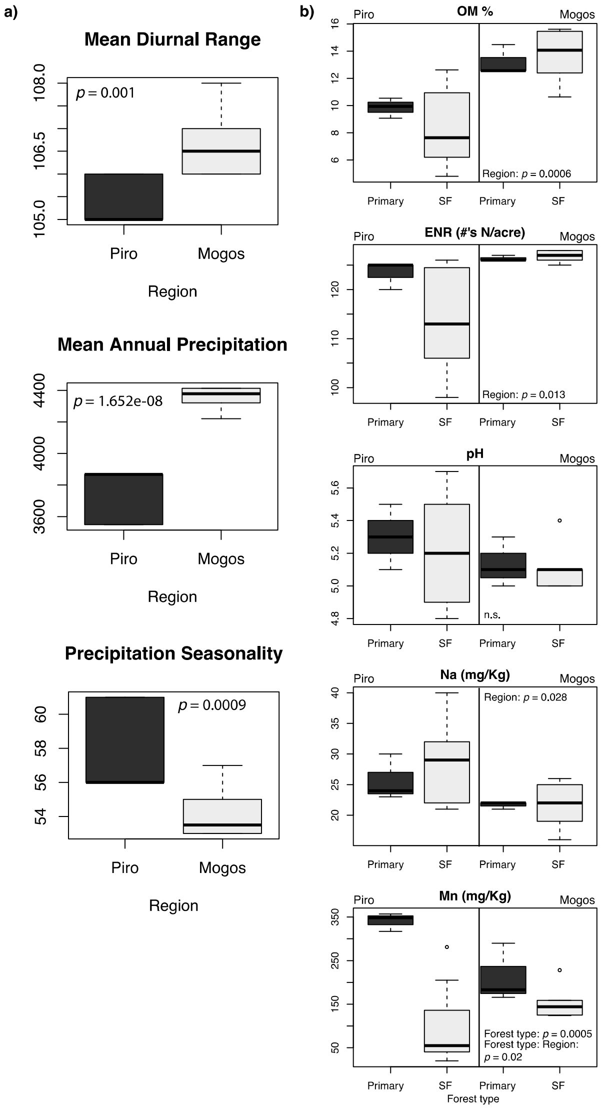

Figure S2. Global nonmetric multidimensional scaling (GNMDS) analysis of the functional groups dataset. Environmental (climatic and edaphic) and tree family abundance with significant correlation with the ordination axes are displayed. Abbreviations: MAP: mean annual precipitation, MDRT: mean diurnal range of temperature, PS: precipitation seasonality.

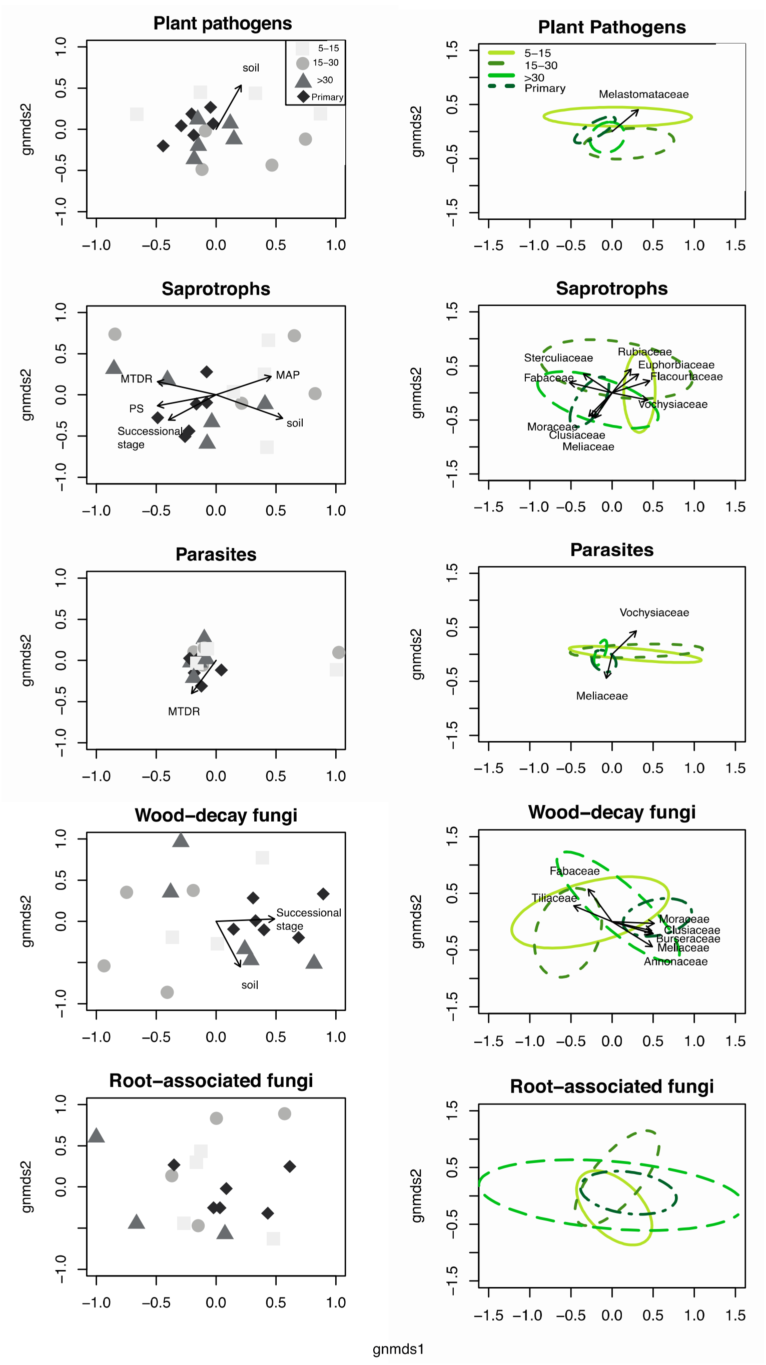

Table S1. Plot number, site information, forest stand age and geographic coordinates of the 19 permanent forest plots sampled for fungal community analyses.

Sample	Plot	Site	Stand age	Latitude	Longitude
CR 1	1	Piro - La U	15-30	8.414521	-83.349166
CR 2	2	Piro – Laguna silvestre	Primary	8.411087	-83.339610
CR 3	3	Piro – Osa Friends	Primary	8.408313	-83.335120
CR 4	4	Piro – Los higuerones	30<	8.406355	-83.337594
CR 5	5	Piro - Terciopelo	15-30	8.402265	-83.341205
CR 6	6	Piro - Río	30<	8.400080	-83.337995
CR 7	7	Piro -BK	5-15	8.396825	-83.306056
CR 8	8	Piro – Manuel Ramírez	Primary	8.403934	-83.307442
CR 9	9	Piro - Lapa Ríos	15-30	8.391543	-83.295043
CR 10	10	Mogos – Bahia Chal	Primary	8.761829	-83.467603
CR 11	11	Mogos - Iglesia	30<	8.764016	-83.366803
CR 13	13	Mogos - Primario	Primary	8.754686	-83.373081
CR 14	14	Mogos Helechal	5-15	8.754464	-83.366760
CR 15	15	Piro - Terciopelo	30<	8.401407	-83.342033
CR 16	16	Piro – Manuel Ramírez	30<	8.393010	-83.322035
CR 17	17	Mogos - Pavona	5-15	8.782129	-83.415306
CR 18	18	Mogos - Pavona	15-30	8.785882	-83.421489
CR 19	19	Mogos	Primary	8.755942	-83.370466
CR 20	20	Mogos	5-15	8.783701	-83.420093

Table S2. Fungal OTUs considered as significant indicators of the successional stages of regenerating tropical forest, with corresponding *p*-values, assigned functional guild, matching Species Hypothesis, ITS2 rDNA sequence similarity (%) and taxonomic classification of the most similar matching sequence in the UNITE+INSD dynamic Species Hypotheses database (version released on October 10, 2017).

OTU ID	*Forest Type*	*p Value*	*Accession*	*%*	*bp*		*Order*	*Phylum*
OTU 2088	5-15	0.014	AM260868	96	200	SH474962.07FU	Mortierellales	Mortierellomycota
OTU 1905	15-30	0.015	UDB020569	73.1	182	SH026232.07FU	Coniochaetales	Ascomycota
OTU 350	15-30	0.016	EU686753	75.9	203	SH030917.07FU	Sordariales	Ascomycota
OTU 23	15-30	0.048	JN890293	80.5	200	SH468137.07FU	Sordariales	Ascomycota
OTU 631	15-30	0.018	KP013012	97.5	200	SH190270.07FU	Agaricales	Basidiomycota
OTU 603	<30	0.018	KX347480	91.5	200	SH174595.07FU	unknown	unknown
OTU 246	Primary	0.009	GU187992	99	193	SH480688.07FU	Botryosphaeriales	Ascomycota
OTU 334	Primary	0.01	KU314949	96.3	190	SH524979.07FU	Pleosporales	Ascomycota
OTU 150	Primary	0.013	KU534808	100	200	SH630161.07FU	unknown	0
OTU 926	Primary	0.031	FJ612616	93.7	189	SH211130.07FU	unknown	Ascomycota
OTU 1020	Primary	0.043	EF423546	97.5	200	SH217133.07FU	Xylariales	Ascomycota
OTU 1378	Primary	0.046	KM374325	74.6	201	SH495740.07FU	unknown	Ascomycota
OTU 922	Primary	0.048	KP235767	88.1	201	SH521485.07FU	Venturiales	Ascomycota
OTU 847	Primary	0.035	FN812805	89.7	203	SH211372.07FU	unknown	Ascomycota
OTU 2634	Primary	0.04	AF502896	93.8	194	SH200172.07FU	Xylariales	Ascomycota
OTU 1377	Primary	0.042	FJ612763	97	202	SH195305.07FU	Sordariales	Ascomycota
OTU 1198	Primary	0.046	KT328673	95.7	186	SH492885.07FU	Pleosporales	Ascomycota
OTU 1498	Primary	0.047	KM265826	99.4	170	SH182681.07FU	Hypocreales	Ascomycota
OTU 1227	Primary	0.042	KF435425	70.5	200	SH181517.07FU	unknown	Ascomycota
OTU 1224	Primary	0.043	JQ838232	100	200	SH213908.07FU	Xylonomycetales	Ascomycota
OTU 1380	Primary	0.048	HM239729	88.1	201	SH181605.07FU	Archaeorhizomycetales	Ascomycota
